# Key physiological indicators and technological trends in physiology-directed cardiopulmonary resuscitation: A narrative review

**DOI:** 10.1016/j.resplu.2025.101180

**Published:** 2025-11-28

**Authors:** Pengfei Zhao, Yali Tong, Zifan Du, Shuai Ma, Bin Fan

**Affiliations:** School of Disaster and Emergency Medicine, Tianjin University, Tianjin 300000, China

**Keywords:** Physiology-directed cardiopulmonary resuscitation, Physiological indicators, Noninvasive monitoring, Closed-loop cardiopulmonary resuscitation, Personalized resuscitation

## Abstract

Cardiac arrest remains a critical public health challenge with consistently low survival rates worldwide, underscoring the urgent need to optimize the quality of cardiopulmonary resuscitation. Physiology-directed cardiopulmonary resuscitation, through real-time monitoring of parameters such as end-tidal carbon dioxide and diastolic blood pressure, enables clinicians to objectively assess cardiopulmonary resuscitation quality and make adjustments in real time, advancing towards personalized resuscitation therapy. This review investigates the relationships between key physiological indicators – including coronary perfusion pressure, arterial blood pressure, end-tidal carbon dioxide, and cerebral regional oxygen saturation – and compression parameters, along with their value for resuscitation outcomes. Moreover, it synthesizes current evidence supporting the efficacy of physiology-directed resuscitation. The review also addresses the limitations of invasive monitoring techniques and explores the application of noninvasive methods in cardiopulmonary resuscitation, providing critical analysis and insights into emerging technological trends in physiology-directed resuscitation.

## Introduction

Cardiac arrest (CA) is a sudden circulatory failure syndrome that is potentially reversible, yet time-critical. Its fundamental pathological feature is the abrupt cessation of cardiac mechanical activity, resulting in a systemic interruption of blood flow and oxygen supply. If not intervened upon within the critical time window, it rapidly progresses to irreversible multiple organ failure, particularly involving oxygen-sensitive organs such as the brain and heart. Global annual incidence rates of out-of-hospital cardiac arrest (OHCA) treated by emergency medical services (EMS) are estimated to range from 30.0 to 100.2 per 100,000 population; only 3.1–20.4 % of OHCA patients survived to hospital discharge or 30 days.^1^ The annual incidence of EMS-treated OHCA in Europe is 55 cases per 100,000 residents, with a survival rate of 7.5 %; meanwhile, the annual incidence of in-hospital cardiac arrest (IHCA) ranges from 1.5 to 2.8 cases per 1,000 hospital admissions, with survival ranging from 27.3 % to 62 %.^2^ The incidence of EMS-treated OHCA in the United States is 83.4 per 100,000 people,^3^ with a survival-to-discharge rate of 10.5 %.^4^ Meanwhile, approximately 292,000 IHCA cases occur annually,^3^ with a 23.6 % survival to hospital discharge.^4^ The annual number of reported OHCA cases in China is approximately 1.05 million, with a survival-to-discharge rate of only 1 %.^5^ For IHCA, the incidence stands at 8.4 cases per 1,000 hospitalized patients, and the survival-to-discharge rate reaches 9.4 %.^6^.

Cardiopulmonary resuscitation (CPR), involving chest compressions (CC), ventilation, and defibrillation,^7^ is the most direct and effective method to facilitate return of spontaneous circulation (ROSC) and salvage CA patients. Nevertheless, the quality of resuscitation efforts is the primary factor determining patient prognosis, and suboptimal CPR significantly reduces ROSC and survival rates.^8^ To enhance the quality of CPR, audiovisual feedback devices have been implemented in both in-hospital and pre-hospital resuscitation settings to enable real-time correction of CC rate and depth.^8^ Metronomes have the capacity to standardize CC rate, while force/acceleration sensors offer dual feedback on depth and rate.^9^ Nevertheless, factors such as CC hand position also critically influence resuscitation quality.^10^ Therefore, the existing audiovisual feedback systems are deficient in their ability to comprehensively assess and provide guidance on these aspects.^9^ Furthermore, the International Liaison Committee on Resuscitation (ILCOR) International Consensus emphasizes that the primary goal of CPR is to restore adequate organ perfusion,^11^ not merely achieve mechanical targets. Audiovisual feedback focuses on mechanical parameters, such as compression depth and rate,^12^ largely ignoring the patient's physiological response to resuscitation efforts.

Recently, physiology-directed CPR has emerged as a research focus. While the concept of physiology-directed resuscitation lacks a universally accepted definition, it advocates for the real-time monitoring of key indicators (such as coronary perfusion pressure (CPP), diastolic blood pressure (DBP), and end-tidal carbon dioxide (ETCO_2_))^13^, ^14^ to dynamically evaluate and optimize the quality of resuscitation efforts. Through immediate, objective feedback on the effectiveness of each compression, this approach helps to improve upon the “one-size-fits-all” approach of standardized protocols. Specifically, low ETCO_2_ or DBP values can alert rescuers to suboptimal compressions, prompting immediate adjustments in technique and enabling real-time assessment of the intervention's effect. On a more fundamental level, physiology-directed resuscitation has the potential to satisfy the CA patient's demand for CPP and blood flow, a crucial determinant for the survival of the heart and brain.

Some pre-clinical studies suggest potential modest benefits of CPR strategies targeting hemodynamic indicators or ETCO_2_, indicating associations with improved ROSC rates and short-term survival.^15^, ^16^ A systematic review suggests potential benefits of physiology-directed CPR over traditional mechanics-directed protocols.^17^ Physiology-directed resuscitation may constitute a possible pathway for enhancing resuscitation quality and patient outcomes in the future.^17^ This review focuses on physiological indicators during adult CPR. It comprehensively discusses the associations between these indicators and resuscitation parameters, reviews the progress in using them to guide resuscitation, and also examines advancements in related technologies such as emerging noninvasive monitoring techniques.

## Physiological indicators for guiding CPR

The 2025 American Heart Association (AHA) Guidelines for CPR and Emergency Cardiovascular Care explicitly includes DBP and ETCO_2_ in its recommended categories of physiology-directed indicators.^18^ In recent years, increasing research has recognized the growing value of additional indicators beyond these two, such as cerebral regional oxygen saturation (rSO_2_), for guiding CPR. To evaluate the application value of existing physiological indicators and supplement details not fully addressed in the guidelines, this review summarizes current research on CPR physiological indicators from key dimensions (e.g. correlation with CC parameters, predictive value for ROSC). Specifically, we examine the relationships between various physiological indicators and both compression quality and ROSC, subsequently discussing threshold values for these indicators. More importantly, this chapter compiles existing research on physiology-directed resuscitation and discusses related technical issues. For this chapter, our primary inclusion criteria focused on data from adult human or animal studies. Data from pediatric human and animal models were only incorporated under two conditions: first, when adult data were unavailable; second, when high-quality pediatric data could substantively enrich our discussion of physiological indicators.

### CPP

CPP, defined as aortic diastolic pressure minus right atrial diastolic pressure,^19^ is considered the gold-standard physiological indicator for assessing myocardial blood flow efficiency.^20^ Its magnitude is jointly associated with compression depth, rate, and continuity.^21^, ^22^, ^23^, ^24^, ^25^ Deeper and faster compressions steepen the aortic–right-atrial gradient and raise CPP,^22^, ^23^, ^24^ whereas even brief pauses rapidly drive CPP below the critical perfusion threshold.^25^ Thus, CPP reflects how well compression depth, rate and continuity are adapted to coronary perfusion demands; maintaining this match is essential for optimizing myocardial blood flow.

CPP is a predictor of ROSC: survivors consistently maintain higher CPP throughout resuscitation across species.^26^, ^27^ To standardize CPP-directed resuscitation, the 2010 AHA Guidelines set 15 mmHg as the minimum threshold^28^; three years later, an AHA consensus statement raised this target to 20 mmHg to further improve outcomes.^14^

Hemodynamic-directed CPR (HD-CPR) elevates CPP from a monitoring indicator to a dual monitoring-therapeutic target. Its core strategy combines systolic blood pressure (SBP)-titrated compression depth with vasopressor-mediated CPP maintenance. Animal studies suggest potential benefits of HD-CPR.^15^, ^29^, ^30^, ^31^, ^32^, ^33^ These potential benefits manifest in three aspects: (1) HD-CPR groups universally exhibit elevated CPP (mean increase 21–28 mmHg; *P* ≤ 0.03)^15^, ^29^, ^30^, ^33^; (2) reduced compression depth (4.0–10.0 mm decrease, *P* < 0.05)^15^, ^30^, ^31^, ^32^; and (3) improved survival rates – including 2.7–6.0-fold relative increase at 45 min (*P* = 0.002),^15^, ^31^ 62.5–80 % absolute increase at 24 h (*P* ≤ 0.03).^29^, ^32^ The relationship between compression depth, CPP, and survival presents a counterintuitive finding: deeper compressions (within guideline limits of ≤6 cm) do not necessarily confer greater CPP or higher survival rates. The underlying mechanism may involve two factors. On one hand, HD-CPR's individualized perfusion–compression coupling optimizes resuscitation quality through hemodynamic demand–compression intensity matching. On the other hand, these animals received more frequent vasopressor administration on average, which not only elevated CPP and SBP but also enabled shallower CC. Collectively, these preclinical studies lend partial support to personalized resuscitation strategies wherein physiology-directed dynamic adjustment of compression parameters combined with vasopressor therapy may prove superior to conventional fixed-depth approaches, thereby improving resuscitation outcomes.

While CPP offers some value in guiding resuscitation, its invasive monitoring requirements and suboptimal timeliness limit practical implementation. Measuring CPP requires simultaneous intra-arterial and right atrial pressure monitoring via catheters – a procedure with critically low feasibility in OHCA settings and is therefore not recommended for routine clinical use.^14^ Even in in-hospital environments, catheter placement averages 16.5 ± 6.0 min, substantially exceeding the critical intervention window.^19^

### Arterial blood pressure

Arterial blood pressure, as a direct hemodynamic reflection during CPR, exhibits dynamic changes closely associated with CC parameters. It can also serve to identify ROSC during CC.^11^, ^28^, ^34^ SBP primarily reflects peak flow generated by compressions, while DBP more closely approximates the diastolic component of CPP. Together, they provide complementary value in resuscitation quality assessment.

SBP can to some extent reflect compression depth, force, and rate.^35^, ^36^, ^37^, ^38^ In a pediatric study, SBP rises with deeper compressions—beyond approximately 38 mm, each 10-mm increase was associated with a >15-mmHg elevation in SBP.^35^, ^38^ A study by Sainio et al. identified that achieving optimal SBP levels necessitates synergistic rate-depth targeting, specifically 100–120 compressions per minute combined with 50–60 mm depth to reach the target threshold of ≥85 mmHg.^36^ Current evidence remains insufficient to establish SBP as a robust and independent resuscitation feedback indicator. Its role in resuscitation feedback is as an adjunct indicator for CPP-directed resuscitation.

DBP demonstrates limited correlation with compression parameters,^36^, ^38^ yet serves as a surrogate for CPP with superior predictive utility for ROSC and other prognostic outcomes compared to SBP.^39^ DBP thresholds demonstrate prognostic value across experimental and clinical settings. Pediatric evidence primarily reveals age-stratified thresholds: ≥25 mmHg in infants, ≥30 mmHg in children correlate with improved survival and neurological outcomes.^40^, ^41^ The 2025 AHA Guidelines for Pediatric Advanced Life Support confirm that, with continuous invasive arterial blood pressure monitoring, healthcare professionals may set DBP targets at ≥25 mmHg for infants and ≥30 mmHg for children aged one year and older.^42^ Similarly, the European Resuscitation Council (ERC) Pediatric Life Support Guidelines specify targeting an intra-arterial DBP of ≥25 mmHg for infants under 1 year old and ≥30 mmHg for children between 1 and 18 years old, provided that intra-arterial blood pressure monitoring was already established at the time of CA.^43^ Additionally, regarding adult data, a clinical retrospective study identified DBP as a potential clue for target-directed resuscitation in adults with OHCA. The study reported a DBP threshold of 35 mmHg as beneficial for achieving ROSC.^44^ A clinical study by Kim et al. found that DBP served as a prognostic marker for predicting the likelihood of achieving sustained ROSC, and further revealed that during resuscitation, follow-up DBP values >27 mmHg and delta DBP values >7 mmHg were associated with the attainment of sustained ROSC.^45^ The newly released ERC guidelines recommend targeting a DBP of ≥30 mmHg for adults when performing physiology-directed CPR using arterial blood pressure monitoring.^13^

The advantage of arterial pressure monitoring lies in providing real-time hemodynamic guidance.^46^ Therefore, patients with established continuous invasive arterial blood pressure monitoring can use it to assess CPR quality.^18^ In a substantial proportion of out-of-hospital settings, performing invasive blood pressure monitoring is likely to remain a challenge.^13^, ^18^, ^47^ Thus, while arterial pressure remains vital for CPR quality assessment, its application requires contextual consideration (in-hospital vs. out-of-hospital), resource availability, and patient factors. Future research should prioritize noninvasive DBP prediction models.

### ETCO_2_

ETCO_2_ maintains a highly linear correlation with cardiac output (CO),^48^, ^49^ a relationship that persists during CPR,^50^ making it a surrogate indicator for CO during resuscitation.

ETCO_2_ exhibits quantifiable associations with CPR quality parameters, establishing its role as a physiological feedback marker.^51^ A clinical observational study by Sheak et al. found that for every 10-mm increase in compression depth, ETCO_2_ increased by an average of 1.4 mmHg (*p* < 0.001).^52^ Similarly, a clinical retrospective analysis by Ruiz et al. described a comparable relationship—where an incremental depth of 10 mm was associated with a 2.6 mmHg elevation in ETCO_2_.^53^ Murphy et al. also reported that a 10-mm increase in compression depth was associated with a 4.0 % increase in ETCO_2_ (*p* < 0.0001).^54^ These studies preliminarily indicate a positive correlation between ETCO_2_ and compression depth. Additionally, Sheak et al. and Murphy et al. investigated the relationship between ventilation rate and ETCO_2_ in their respective studies. Their findings indicated that for every 10 breaths/min increase in rate, ETCO_2_ decreased by 3.0 mmHg and 17.4 %, respectively.^52^, ^54^ No statistically significant association exists with compression rate.^51^, ^53^

The threshold criteria for ETCO_2_ in predicting ROSC have evolved through two distinct phases. Early research established an association between ETCO_2_ > 10 mmHg and increased likelihood of ROSC.^55^, ^56^ Based on accumulating evidence, ETCO_2_ ≥ 20 mmHg is now widely used as a predictor of ROSC.^57^, ^58^, ^59^, ^60^, ^61^ The latest edition of the AHA guidelines further confirms this threshold.^18^ The ERC guidelines propose a more ambitious target: achieving an ETCO_2_ greater than 25 mmHg during physiology-directed CPR.^13^

ETCO_2_-directed individualized resuscitation has been initially carried out in pediatric models. Compared to standard CPR, the ETCO_2_-directed approach achieves higher ETCO_2_ levels and demonstrates a more than 3-fold improvement in resuscitation success rates.^16^, ^62^ The physiological advantage resides in real-time ETCO_2_ feedback, which helps facilitate the precise optimization of resuscitation quality.

Consequently, ETCO_2_ demonstrates tripartite potential utility throughout CPR: (1) real-time quality feedback: quantifying compression depth/ventilation efficacy; (2) predictive target: ≥20 mmHg as a ROSC predictor; (3) individualized intervention: dynamically guiding compression parameters and pharmacotherapy timing. Its noninvasive real-time monitoring capabilities and promising guiding value make it one of the candidate indicators for future clinically applicable physiology-directed resuscitation. More importantly, it is applicable in both out-of-hospital settings (e.g. ambulances, on-site resuscitation) and in-hospital settings, and has been recognized as the current highly valuable tool for evaluating CPR quality.^63^

### Cerebral rSO_2_

Cerebral rSO_2_, monitored noninvasively in real-time via near-infrared spectroscopy (NIRS), provides unique insights into cerebral oxygenation and resuscitation quality during CA.^64^ Studies demonstrate that cerebral rSO_2_ is sensitive to cerebral hemodynamic changes induced by CC.^65^, ^66^, ^67^

Cerebral rSO_2_ demonstrates predictive value for ROSC, validated across systematic reviews, meta-analyses, and observational studies.^68^, ^69^, ^70^ Patients achieving ROSC exhibit substantially higher cerebral rSO_2_ compared to non-ROSC counterparts, with an absolute elevation exceeding 10 %.^71^, ^72^, ^73^ Critically, cerebral rSO_2_ values predominantly surpass 40 % in survivors,^69^, ^70^, ^72^ whereas thresholds <26 % predict near-universal resuscitation failure.^68^ Furthermore, ΔrSO_2_ provides dynamic predictive utility: an increase ≥15 % strongly associates with ROSC.^68^, ^74^

Cerebral rSO_2_ monitoring provides a tool for continuous cerebral oxygenation assessment during CA,^65^ delivering feedback on resuscitation quality.^75^ Crucially, its predictive efficacy for ROSC is supported by partial evidence, demonstrating advantages over ETCO_2_. For example, Engel et al. identified cerebral rSO_2_ as a superior predictor of ROSC compared to ETCO_2_.^74^ Putowski et al. further confirmed through area under the curve (AUC) analysis that cerebral rSO_2_ outperforms ETCO_2_ in ROSC prediction (AUC = 0.978 vs. 0.815; *P* < 0.001).^71^ Despite advantages of rapid deployment and noninvasive continuous monitoring, technical limitations warrant acknowledgment.^76^ Ambient light interference,^77^ skin pigmentation variations,^78^ and device heterogeneity partially^79^ explain threshold discrepancies across studies. Nevertheless, the rapid response capability and cerebral oxygenation specificity conferred by NIRS technology during CPR endow cerebral rSO_2_ with considerable translational potential for guiding individualized resuscitation strategies.

### Peripheral oxygen saturation (SpO_2_)

Evidence regarding SpO_2_ as a feedback indicator for resuscitation quality or as a predictor of ROSC remains limited.^80^, ^81^, ^82^ SpO_2_ changes correlate with compression quality, specifically reflecting optimal hand positioning and force intensity. One experimental finding indicates that it can predict ROSC: patients achieving SpO_2_ ≥ 94 % during CPR are more likely to achieve ROSC, while those with unmeasurable readings experience resuscitation failure.^80^ Moreover, in OHCA scenarios, ΔSpO_2_ exhibits predictive capability for ROSC earlier than ΔrSO_2_.^82^ However, it should be noted that these conclusions are currently derived from a limited number of studies and have not yet been robustly validated or supported by broader research.

In contrast, photoplethysmography (PPG) transcends its conventional application in pulse oximetry by leveraging raw waveform characteristics to correlate amplitude with compression depth, CPP, and ETCO_2_,^83^ while enabling real-time extraction of compression rates^84^, ^85^ and prediction of ROSC.^86^, ^87^, ^88^, ^89^

Significantly, current evidence is insufficient to establish SpO_2_ as a reliable indicator for guiding CPR optimization. Moreover, the accuracy of SpO_2_ measurements during CPR has been questione.^43^, ^90^ Despite these limitations, further collective investigations into the correlation between peripheral oximetry (including its waveform characteristics) and CPR is encouraged, necessitating involvement from multiple research groups.

## Technologies enabling physiology-directed CPR: Current trends

Current CPR research frontiers focus on noninvasive monitoring and intelligent closed-loop systems, addressing limitations of traditional approaches: invasive methods lack timeliness and feasibility during arrest, while static thresholds fail to meet dynamic optimization needs. This drives three pivotal advances – emerging noninvasive monitoring, artificial intelligence (AI), and closed-loop CPR executing clinician-supervised sense-decide-actuate cycles. Unlike previously discussed mature noninvasive monitoring techniques such as ETCO_2_ or rSO_2_, the first subsection explores innovative noninvasive monitoring methods currently under investigation, reflecting emerging developments in noninvasive monitoring technology. Subsequently, discussions surrounding AI and closed-loop CPR aim to illustrate their potential within the field, thereby highlighting potential directions for future optimization of resuscitation practices.

### Emerging noninvasive monitoring techniques

This section scrutinizes several emerging noninvasive monitoring techniques. Such technologies possess two defining characteristics: Firstly, they embody the redevelopment and adaptive repurposing of existing technologies; secondly, they are oriented toward establishing indirect mappings of invasive indicators or uncovering substitute physiological indicators.

Impedance cardiography (ICG) exemplifies signal repurposing in resuscitation science: originally designed for noninvasive CO measurement,^91^, ^92^, ^93^, ^94^ ICG has the potential to become a CPR quality feedback tool. Its amplitude correlates with compression depth,^95^, ^96^, ^97^, ^98^, ^99^ while its waveform characteristics reflect compression rate^100^ and ventilatory events.^101^ Crucially, ICG demonstrates significant correlations with CO^102^, ^103^ and ETCO_2_^102^, ^104^ during CPR. This signal-to-physiology mapping holds promise for replacing invasive monitoring, though inter-subject variability remains the primary translational challenge. The integration of deep learning and multimodal signal fusion represents a promising approach to address inter-subject variability in ICG measurements, though further investigation is required to validate this strategy.

Carotid blood flow velocity correlates strongly with CO.^105^ With technological advancements, the monitoring of carotid blood flow velocity is evolving from ultrasound towards hands-free Doppler and quantitative patch devices.^106^, ^107^, ^108^ Continuous carotid blood flow velocity measurement during CPR enables identification of optimal versus suboptimal CC positions.^108^ Crucially, Zhao et al. demonstrated a strong positive correlation between velocity–time integral (VTI) and compression depth, with 30 cm VTI emerging as the optimal survival threshold.^106^

In conclusion, the described technologies still require further validation but have preliminarily demonstrated potential feasibility. Their evolution toward fully noninvasive and wearable form represents a trajectory warranting attention.

### The application potential of AI in CPR

AI represents a promising frontier in CPR, with multifaceted applications under active investigation.^109^ Research demonstrates that AI has the potential to: predict CA,^110^ optimize CPR quality,^111^, ^112^ predict ROSC without interrupting CC,^112^, ^113^ and prognosticate neurological outcomes.^114^, ^115^ Early detection of impending IHCA contributes to improved clinical outcomes. Lee et al. developed a multimodal machine learning algorithm using the Multiparameter Intelligent Monitoring of Intensive Care (MIMIC)-IV database, with external validation conducted using the Electronic Intensive Care Unit Collaborative Research Database (eICU-CRD) and National Taiwan University Hospital databases; their model showed it could identify trajectories of clinical deterioration up to 13 h in advance.^110^ Furthermore, reducing pauses in CC represents a key requirement for optimizing resuscitation. Addressing this, Isasi et al. created a machine learning architecture capable of rhythm identification during CC without requiring their interruption, thereby minimizing no-flow time due to CPR breaks and enhancing overall CPR quality.^116^ Additionally, predicting CA outcomes carries significant implications for therapeutic decisions and the determination of life-sustaining treatment. In this context, Kwon et al. developed and validated a deep learning-based prognostic system for OHCA that accurately predicted neurological recovery and survival to hospital discharge.^114^

Furthermore, studies are exploring how AI can infer physiological indicators through compression waveform analysis, establishing correlations between waveform characteristics and indicators such as carotid blood flow and arterial pressure.^117^, ^118^, ^119^ Moving beyond waveform decoding, AI has shown early promise in noninvasively reconstructing invasive physiological indicators through multimodal signal fusion, as exemplified by the prediction of CPP via integrated electrocardiogram and PPG analysis.^120^

The integration of AI into resuscitation protocols, while still primarily in the research domain, could signify a future shift. However, the reliability of AI algorithms correlates directly with the quality of their training data. When data are unreliable, incomplete, or biased, model predictions may become inaccurate or even harmful.^121^ A potential challenge in deploying AI for actual resuscitation scenarios lies in model interpretability and trustworthiness. AI models are often characterized as “black boxes”, because their decision-making processes cannot be fully understood.^122^ This lack of transparency critically erodes clinician and patient trust in AI systems.^121^ Therefore, the next critical step may need to pioneer explainable edge AI that provides real-time signal analysis and transparent decision feedback. Delivering human-understandable AI constitutes a fundamental objective for its own decision-making utility,^123^ helping address trust, safety, and ethical concerns in high-risk medical scenarios. Simultaneously, this represents a vital aspect of facilitating interaction and collaboration between dual intelligences to enhance CPR quality.

### Closed-loop mechanical CPR system

**Fig. 1:**
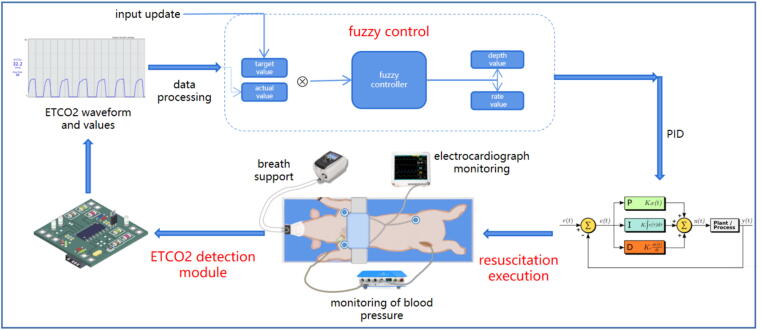
Closed-loop mechanical CPR system architecture with ETCO_2_ feedback. This diagram illustrates a closed-loop CPR system using ETCO_2_ as the core physiological feedback to optimize CA resuscitation.

Integrating real-time feedback mechanisms into CPR workflow helps improve resuscitation quality.^124^ Mechanical CPR devices possess the potential for integrated fusion with physiological monitoring technologies^125^ and intelligent decision-making algorithms, which aids in constructing a closed-loop mechanical CPR system with sense-decide-actuate capabilities. Such a system uses physiological signals (such as ETCO_2_ and cerebral rSO_2_) as input, continuously quantifying the physiological effectiveness of resuscitation, and then uses intelligent algorithms to dynamically optimize CPR parameters such as depth and rate – potentially serving as a possible option for achieving personalized, physiology-directed resuscitation (Fig. 1).

Several studies are investigating the efficacy of closed-loop CPR systems through animal experiments, contributing to a deeper understanding of physiology-directed resuscitation.^120^, ^122^, ^126^ In 2018, Suh et al. first developed an ETCO_2_-directed closed-loop robotic CPR system that explored optimal CC positions. While this system did not significantly improve ROSC rates in porcine CA models, it nevertheless demonstrated significant improvement in neurological deficit scores at 48 h post-ROSC compared to manual CPR.^127^ 2020, Sebastian et al. evaluated the feasibility of using a closed-loop machine-controlled CPR (MC-CPR) system to optimize CPP. In their system, real-time CPP measurements were fed back to a control algorithm, which adjusted compression/decompression characteristics accordingly. Comparative experiments indicated that MC-CPR improved hemodynamics during 30 min of CPR administration.^128^ Collectively, these investigations demonstrate that the core principle underpinning closed-loop mechanical CPR systems is the integration of clinically meaningful feedback signals (such as CPP, ETCO_2_) to drive adaptive adjustments of resuscitation parameters – ultimately aimed at optimizing CPR outcomes.

The goal of closed-loop resuscitation is to dynamically identify and execute the optimal resuscitation strategy tailored to the individual patient, guided by physiological monitoring. Thus, two factors constrain closed-loop resuscitation: the choice of physiological monitoring technology, and the design of decision-making algorithms. The ideal physiological monitoring technology should be noninvasive and sufficiently clinically reliable. Decision-making algorithms involve balancing exploration (trying different strategies and collecting physiological data) and exploitation (executing the currently known best strategy), while also profoundly influencing the level of trust between the closed-loop resuscitation process and clinicians. An ideal decision-making algorithm must achieve a delicate balance between speed in identifying the optimal strategy and transparency of the decision-making process. Although the transition of closed-loop mechanical CPR systems to clinical application appears promising, there is undoubtedly a long road ahead.

## Discussion

Current resuscitation protocols according to AHA or ERC guidelines mandate CC depths of 5–6 cm at rates of 100–120 compressions/min. However, real-world CPR implementation demonstrates significant guideline non-compliance, predominantly manifested as inadequate CC depth, which impairs physiological outcomes. Furthermore, standardized protocols may obscure resuscitation variations across global regions and ethnicities, proving suboptimal for anatomically specific populations (e.g. obese or cachectic patients).^129^, ^130^, ^131^, ^132^ A shift toward patient-centered care – involving the monitoring and optimization of individual physiological responses to CPR – may improve ROSC and survival rates.

Current evidence identifies ETCO_2_ and cerebral rSO_2_ as promising noninvasive physiological indicators during CPR. Invasive techniques for measuring CPP are impractical due to procedural complexity and time constraints. SpO_2_ currently lacks sufficient evidence for CPR quality feedback, while invasive blood pressure may serve as a supplementary indicator. The convergence of emerging noninvasive monitoring technologies, AI, and closed-loop resuscitation represents the frontier of physiology-directed CPR, heralding a paradigm shift toward personalized and intelligent precision resuscitation.

Indicators of physiology-directed resuscitation have been previously reviewed by Bray et al.^133^ and Bernard et al.^134^ with similar findings. However, we provide a more comprehensive discussion of valuable physiological indicators. Furthermore, our coverage of emerging noninvasive monitoring techniques, AI, and closed-loop CPR may represent one of the cutting-edge reviews of technological advancements in this domain.

It must be recognized that resuscitation constitutes a systematic endeavor reliant on collaboration. Modern CPR has emerged through the integration of CC, defibrillation, and ventilation.^7^ Efforts to improve resuscitation quality are multifaceted, where ventilation and defibrillation are all indispensable components requiring integrated implementation. In resuscitation practice, epinephrine has also been consistently recommended for the treatment of CA.^135^ As a vasoconstrictor, epinephrine increases blood flow to the heart and cerebral cortex by elevating systemic vascular resistance,^59^, ^136^, ^137^ raises blood pressure (particularly DBP), and enhances CPP (which is a critical factor for achieving ROSC).^138^, ^139^

However, the critical question remains: Does current physiology-directed resuscitation provide tangible and practical guidance for clinical practice? Undeniably, substantial knowledge gaps persist. The reality is that physiology-directed resuscitation holds a certain degree of promise, but several major and fundamental issues must be addressed before it can be considered for clinical implementation.

First, the definition of physiology-directed resuscitation and its practical implementation details have not yet been clearly defined. Preclinical studies conducted by scholars such as Sutton, Morgan, and Hamrick pioneered concepts like adjusting compression depth based on blood pressure or modifying compression parameters to maximize ETCO_2_.^16^, ^32^, ^33^, ^140^ However, the complex protocols employed in these animal models – involving the implementation of CPP monitoring, intricate vasopressor bolus sequences, and continuous parameter adjustments – are invasive and difficult to replicate straightforwardly in clinical resuscitation, particularly in pre-hospital emergency settings. The critical leap from these initial proof-of-concept studies to establishing a feasible, standardized clinical protocol has not yet been achieved.

Second, and more critically, we lack any high-quality clinical evidence demonstrating that physiology-directed resuscitation improves outcomes compared to conventional resuscitation. This evidence gap is multifaceted. Regarding target selection, although DBP and ETCO_2_ are physiologically plausible, the optimal target values for humans remain inadequately defined. In terms of implementation, the most effective methods to achieve these targets remain unclear; for instance, how compression parameters should be adjusted and how the method of vasopressor administration should be selected (i.e., whether blood pressure should be supported through repeated boluses of epinephrine or via continuous infusion). Furthermore, it remains undetermined whether clinical practice should rely on a single indicator or a combination of indicators.

Furthermore, the process of implementing physiological monitoring – such as placing an arterial line for DBP monitoring or performing endotracheal intubation to obtain reliable ETCO_2_ values – may distract the resuscitation team, interrupting the uninterrupted delivery of high-quality CC, precise ventilation, and timely defibrillation. These core components of conventional CPR are interventions with a proven survival benefit. The act of performing physiological monitoring could paradoxically degrade the quality of these fundamental maneuvers, potentially leading to worse outcomes.

Therefore, the current guidance from AHA and ERC is prudent – it acknowledges the feasibility of monitoring DBP and ETCO_2_ but does not yet recommend their routine use for titration.

In summary, although physiology-directed resuscitation shows a certain degree of promise, it remains an incompletely defined preclinical concept. Although animal studies have yielded promising results, we must confront the reality of a severe lack of clinical validation. There is an urgent need to conduct large-scale, rigorous, randomized controlled clinical trials. These trials must not only demonstrate whether physiology-directed resuscitation is efficacious but also determine whether the process of physiological monitoring interferes with conventional resuscitation. In the absence of such critical evidence, physiology-directed resuscitation cannot and should not be advanced into routine clinical application.

## Declaration of generative AI in scientific writing

We utilized DeepL for spelling, grammar, and language refinement. After applying this tool, we reviewed and edited the content as necessary, assuming full responsibility for the final publication.

## CRediT authorship contribution statement

**Pengfei Zhao:** Writing – review & editing, Writing – original draft, Validation, Investigation, Formal analysis, Data curation, Conceptualization. **Yali Tong:** Writing – review & editing, Validation, Conceptualization. **Zifan Du:** Writing – review & editing, Validation, Conceptualization. **Shuai Ma:** Writing – review & editing. **Bin Fan:** Writing – review & editing, Validation, Supervision, Conceptualization.

## Funding

This study was not supported by an external party.

## Declaration of competing interest

We declare that we have no known competing financial interests or personal relationships that could have appeared to influence the work reported in this paper.
